# Effectiveness of shifting traditional lecture to interactive lecture to teach nursing students

**DOI:** 10.17533/udea.iee.v37n1a07

**Published:** 2019-01-06

**Authors:** Ardashir Afrasiabifar, Mosavi Asadolah

**Affiliations:** 1 Nurse, Ph.D. Associate professor, School of Nursing, Yasuj University of Medical Sciences, Yasuj, Iran. email: afrasiabifar.ardashir@yums.ac.ir Yasuj University of Medical Sciences School of Nursing Yasuj University of Medical Sciences Iran afrasiabifar.ardashir@yums.ac.ir; 2 Ph.D candidate. Department Of Medical Education, Medical School, Tehran University of Medical Sciences, Tehran, Iran. email: mousavi.asadolah@yums.ac.ir. Corresponding author. Tehran University of Medical Sciences Department Of Medical Education Medical School Tehran University of Medical Sciences Tehran Iran mousavi.asadolah@yums.ac.ir

**Keywords:** students, nursing, simulation training, lectures, teacher training., estudiantes de enfermería, entrenamiento simulado, clases, formación del profesorado., estudantes de enfermagem, treinamento por simulação, aulas, capacitação de profesores.

## Abstract

**Objective.:**

This study was conducted to examine effectiveness of interactive lecture in teaching nursing students compared to traditional lecture.

**Methods.:**

This study is a quasi-experimental design in which 29 students participated in eighteen sessions of intensive nursing care in Yasuj University of Medical Sciences, Iran. These sessions were randomly allocated for the interactive lecture and the traditional lecture. The interactive lecture consists in this steps: explaining the learning objectives, taking the pre-test, teaching the subjects of each session, Group discussion with introduction of the clinical cases, answering students’ questions and mutual feedbacks, taking the post-test, and introducing students’ future activities. The effectiveness of applied teaching method was evaluated through pre-test, post-test of each session, mid-term and final exams.

**Results.:**

Significant statistical differences were observed in terms of students' mean score (*p*=0.001) and their satisfaction (*p*=0.001) in the interactive teaching method compared to traditional lectures. Further preparation, active participation and received immediate feedback were some benefits reported for the interactive teaching method.

**Conclusion.:**

The interactive lecture resulted in significant learning and furthers nursing students’ active participation in the teaching-learning process.

## Resumen

**Objetivo.:**

Evaluar la efectividad de las clases interactivas para la enseñanza de la enfermería a estudiantes en comparación con las clases tradicionales.

**Métodos.:**

Este estudio es un diseño cuasi-experimental en el que participaron 29 estudiantes en dieciocho sesiones para la enseñanza de cuidados intensivos de enfermería en una universidad en Irán. Las sesiones de las clases interactivas y tradicionales se asignaron al azar. La clase interactiva tenía los pasos: explicar los objetivos de aprendizaje, tomar la prueba previa, enseñar los temas de cada sesión, discutir en grupo los casos clínicos, responder las preguntas de los alumnos y hacer la retroalimentación mutua, realizar la prueba posterior, e introducir las actividades futuras que realizarían los alumnos. La efectividad del método de enseñanza aplicado se evaluó mediante la comparación de la prueba previa con la posterior de cada sesión y de los exámenes intermedios con los finales.

**Resultados.:**

Se observaron diferencias estadísticas significativas en términos de la puntuación media pre y post-test (p<0.001) y de la satisfacción con el método (*p*<0.001) en las clases interactivas en comparación con las clases tradicionales. La preparación adicional, la participación activa y la retroalimentación recibida fueron algunos de los beneficios reportados para el método de enseñanza interactivo.

**Conclusión.:**

En los estudiantes de enfermería la clase interactiva produjo aprendizaje significativo y fomentó la participación activa en el proceso de enseñanza-aprendizaje.

## Resumo

**Objetivo.:**

Avaliar a efetividade das aulas interativas para o ensino da enfermagem a estudantes em comparação com as aulas tradicionais.

**Métodos.:**

Este estudo é um desenho quase-experimental no qual 29 estudantes participaram em dezoito sessões para o ensino de cuidados intensivos de enfermagem numa universidade no Irã. As sessões das aulas interativas e tradicionais se designaram por azar. A aula interativa tinha os passos: explicar os objetivos de aprendizagem; tomar a prova prévia; ensinar os assuntos de cada sessão; discutir em grupo os casos clínicos; responder as perguntas dos alunos e fazer a retroalimentação mútua; realizar a prova posterior; e introduzir as atividades futuras que realizariam os alunos. A efetividade do método de ensino aplicado se avaliou através da comparação da prova prévia com a posterior de cada sessão e dos exames intermédios com os finais.

**Resultados.:**

Se observaram diferenças estatísticas significativas em termos da pontuação média pré e post-test (*p*<0.001) e da satisfação com o método (*p*<0.001) nas aulas interativas em comparação com as aulas tradicionais. A preparação adicional, a participação ativa e a retroalimentação recebida foram alguns benefícios reportados para o método de ensino interativo.

**Conclusão.:**

Nos estudantes de enfermagem a aula interativa produziu aprendizagem significativo e fomentou a participação ativa no processo de ensino-aprendizagem.

## Introduction

Nowadays, revising teaching methods seems necessary.(1^,^^2^) Teaching method is an important element in the teaching-learning process. Lecture method is the oldest and the most common teaching method that is still employed at universities. Although this method is an appropriate way to transfer information and knowledge, it is not a suitable method for long-term learning.(3) Therefore, educational experts suggest other teaching methods to achieve high levels of learning goals.(4) Interactive teaching methods are a group of teaching methods based on Vygotsky’s socio-cultural learning and social constructivism theories.(5^,^^6^) According to Vygotsky, learning has its basis in interacting with other people. Once this has occurred, the information is then integrated on the individual level.(7) Constructivism consists of learning or knowledge construction emphasizing learners as active participants in understanding their environment and their experiences within that environment.(8) The purpose of training is to develop knowledge, skills and attitudes. In interactive teaching methods, students are allowed to form their own professional skills and behaviors. The benefit of this approach is that students participate in the learning process. This educational method contributes to development of professional communication and collaboration skills and student critical thinking.(9) 

One of the interactive methods is interactive lecture in which small groups of students interact with each other and with the information and materials and the teacher is an organizer and facilitator. The members of the group are responsible for their learning and they are actively engaged in the teaching-learning process. Accordingly, the teacher plays a facilitating role by encouraging students to learn in the group, and providing appropriate feedback to them.(8) However, there are contradictory findings about effectiveness of the lecture method compared to other teaching methods. For example, some studies have reported no differences in term of learning outcomes between the lecture method and other teaching methods.(10^,^^11^) While, some studies have reported better learning outcomes for other methods compared to traditional lectures.(12^,^^13^) Murphy and Sharma in their article state that they do not at all assume that in most cases interactive lecture is necessarily more effective than traditional lecture, but the important point remarkable indications tin some areas. In addition, they are interested in developing research to build a better knowledge base for the characteristics of good lectures and good uses of interaction within lectures.(14)

The main goal of nursing education is to train nurses who can play their role in the professional health team to provide high quality services to society.(15)However, nursing education has many challenges, including increasing advances in technology, increasing changes in healthcare systems, the patients’ safety, and nurses multiple tasks.(16^,^^17^) Therefore, nursing students need to develop critical thinking skills, clinical judgment and decision-making to overcome these challenges.(18) Therefore, successful achievement of the expected learning outcomes and the students’ satisfaction depends on the nursing education methods capable of responding to the challenges of healthcare in the clinical complex environments in the 21st century.(19) As a result, periodic changes in teaching methods in line with existing challenges are inevitable.(20) Nursing education needs to have new, student-centered, and interactive teaching methods particularly for teaching the core and specialized courses. These courses are taught for acquiring skills such as critical thinking, clinical judgmental and decision-making, and problem-solving skills to provide specialized nursing services in the clinical setting. Studies into various pedagogical aspects in nursing education also show considerable attention to the concept of successful lecture, requiring interaction and attendance in the classroom.(21) Although the authors maintain that interactive teaching methods are considered by nursing teachers, their effectiveness has been less investigated in nursing education, particularly in teaching basic and specialized courses. Therefore, the present study aimed to use interactive teaching methods in nursing education to teach basic courses. For this purpose, the credit of intensive nursing cares was selected since this credit is offered in the sixth semester and other specialized courses are prerequisites of this credit. We investigated the ease of interactive lecture in nursing education as well as its effectiveness was compared to traditional lectures. 

## Methods

This study is an equivalent quasi experimental design conducted in the second semester of 2015 academic year at the School of Nursing affiliated with Yasuj University of Medical Sciences (Iran). The credits of intensive nursing cares were taught via traditional lecture and interactive lecture. This study was conducted following approval of the Nursing Educational Group, Educational Development Office (EDO) of nursing school, and Educational Development Center (EDC) of the mentioned university. Eighteen sessions of class were randomly allocated for then two methods of teaching of which 9 sessions were taught in the interactive teaching method as the intervention group, and 9 sessions were taught in the traditional lecture method as the control group. Table 1 shows the design of interactive teaching. All of the credit subjects were taught by the first author of the present article according to the course plan. 

**Table1. The:** stages of interactive teaching method

Phase	Activities	Time
Introduction	Explaining the subject of session and expected learning objectives	3-5 minutes
Taking pre-test	Taking the pre-test	3-5 minutes
First teacher’s presentation	Teaching the first subjects of each session	25-30minutes
First group discussion	Group discussion with introduction of the clinical cases	5-7 minutes
Second teacher’s presentation	Teaching the second subjects of each session	25-30 minutes
Second group Discussion	Group discussion with introduction of clinical cases	5-7 minutes
Taking post-test	Answering students’ questions and mutual feedbacks	3-5 minutes
Feedback & Summarization	Taking the post-test	3-5 minutes
Warm- up activities	Introducing students’ future activities	2-3 minutes

The learning outcomes were evaluated through pre and post-tests in each session, mid-term and final exams containing multiple-choice question, short answer, and true and false questions. Mid-term and final exam sheets were anonymously corrected based on the key answer by a member of the related educational group who was blind to types of teaching methods. Then, the papers were also corrected by the main faculty. The final scores were reported after agreement between the first evaluator (master of the course) and the second evaluator. The students’ satisfaction was assessed through the education evaluation form containing five items known as general satisfaction of teaching method, organizing of teaching method, learning objectives and learning stimulation suggested by the University of California at Los Angeles (UCLA). This evaluation form was anonymously made available to the students prior to the onset of the exam session by the experts of educational affairs. They gave assurance to the students that these forms would not be viewed by the main faculty until recording final scores. The collected data were analyzed using SPSS statistical software package through inferential tests such as the t-test or student’s *t.*

## Results

Twenty-nine nursing students participated with the age range of 22-26 years in this study. By the mean score of last five semesters, eight students (27.6%) had a total mean score ≥ 85 (A), fourteen students (48.3%) had a mean score 75-84.99 (B) and seven students (24.1%) were C with a mean score 60-74.99. Midterm score was considered 50% of total score so that 25% of which was related to the lecture method and the remained 25% was related to the interactive teaching method. Final score also included 50% of total score as 25% of final score for the interactive method and the remained 25% for the traditional lecture. The study findings indicated an increase in the students’ mean scores in both midterm exam and final exams for the credit subjects taught through the interactive teaching method compared to the credit subject taught via the traditional lecture method. Independent t-test showed a statistical significant difference in this regard (*p*=0.001). Significant difference was observed in term of overall satisfaction, learning objectives and learning stimulation of the two teaching methods except organizing of teaching method. In other words, the students' mean scores in terms of satisfaction with the teaching method, learning objectives and learning stimulation for the interactive lecture were more than those in traditional lecture method (Table 2).

**Table 2:** Comparing mean scores of exams and students evaluation for the traditional lecture and interactive teaching

			INTERACTIVE TEACHING (N=29)			TRADITIONAL LECTURE (N=29)				INDEPENDENT T SAMPLE TEST	
METHOD	M±SD	Std. Error	95% CI		M±SD	Std. Error	95% CI		Mean Difference	Std. Error	P-Value
			Lower	upper			Lower	Upper			
MIDTERM SCORE	38.2 ±5.1	0.8	36.4	39.9	33.9±4.6	0.8	32.2	35.5	4.3	1.2	0.001
FINAL SCORE	39.1±3.7	0.7	37.8	40.4	35.2±4.5	0.8	33.6	36.7	3.9	1.1	0.001
TOTAL SCORE	77.3 ±6.8	1.3	74.7	79.9	69.1±	1.1	66.8	71.3	8.2	1.7	0.001
SATISFACTION	4.1 ±0.9	0.1	3.7	4.3	2.5±1.1	0.2	2.1	2.9	1.6	0.3	0.001
ORGANIZING OF TEACHING METHOD	3.7±1	0.1	3.2	4	3.6±1.1	0.2	3.2	3.9	0.1	0.2	0.7
LEARNING OBJECTIVES	4.1±1.1	0.2	3.7	4.5	3±1.1	0.1	2.6	3.4	1.1	0.3	0.001
LEARNING STIMULATION	4.1±0.9	0.2	3.8	4.5	2.4±1	0.2	2	2.7	1.7	0.2	0.001

More understanding, further preparation or pre-teaching study, lack of early fatigue, and immediate feedback were the examples of the educational benefits reported for the interactive lecture by the students (Table 3).

**Table 3:** Students’ viewes about educational benefits of the interactive teaching

Educational benfits	n (%)
More understanding for taught subject matters	25 (86.2%)
Further preparation and pre-teaching study	25 (86.2%)
Immediate feedback	24 (82.7%)
Further acceptance for the comments received from students	23 (79.3%)
Participation in group discussions	23 (79.3%)
Lack of premature fatigue	22 (75.8%)
Further interaction among the students	22 (75.8%)
Group responsibility to learn	21 (72.4%)
More motivation to learn	21(72.4%)
Prolonged retention	20 (33.3%)
Others	15 (68.9%)

## Discussion

In this study, we applied interactive lecture to teach the credit of intensive nursing care. The results showed that interactive lecture resulted in more significant learning and satisfaction compared to traditional lecture. Related studies had reported contradictory results about effectiveness of different teaching methods in the field of nursing education. For example, a positive effect was reported for nursing students’ cognitive skills when it was taught via the simulator method, but no change in self- esteem level was reported between the simulator and the traditional lecture.(22) In this regard, Kohistan and Baghcheghi(23) reported better psycho-social climate of the classroom for team-based learningWhereas, acquiring knowledge in the combined lecture method was reported more than the role playing and e-learning techniques; however, long term learning and satisfaction rate were greater than the lecture method.(24) Purghazian *et al*.(24) examined the impact of e-learning, lecture and role playing methods on acquisition, retention and satisfaction. The results of the study showed that the lecture method was better than knowledge acquisition, and the other two methods were better than the lecture method in knowledge management. The results of this study were inconsistent with of our study results in terms of knowledge acquisition, which may be because we compared a type of lecture to the traditional lecture. 

Safari *et al.* also studied the effect of teaching on two methods of lecture and discussion on students' learning and satisfaction. The results of their study indicated that the mean score of the student assessment test was significantly higher in the discussion method than in the lecture method. The findings are consistent with the results of our study.(25) Michelle *et al.*(26) examined the impact of an active teaching method and a traditional (inactive) method on the students' cognitive outcomes, which, according to quantitative evidence, they concluded that the active teaching approach might have an impact on more positive feedback on students’ learning. The results of this study were also consistent with those of our study. The results of this study showed that in the interactive lecture method, student satisfaction significantly increased compared to the traditional lecture method. Other studies have provided contradictory results regarding students' satisfaction. Missildine *et al.* examined the impact of traditional teaching methods, lectures and lectures capture back-up and flipped classroom on student performance and satisfaction. The results of their study demonstrated that students' satisfaction with traditional lecture was higher than with other methods, which is contradictory to our study results.(27) According to our study, it could be mentioned that interactive lecture had been more effective than the traditional method. Therefore, it could increase satisfaction rate in the students. The result of this study showed that stimulation of learning in interactive lecture was significantly more than traditional lecture. This was different from the study results that reported by Miller and colleagues;(28) however, it is in line with the results of the study of Fyrenius.(29) 

The results of this study may be a stimulus for our colleagues to teach using interactive lecture, but the authors of the article argue that teaching-learning is a complex process in which many factors are involved. In particular, students have many differences in terms of their individual characteristics. Therefore, such studies need to be interpreted with more caution. Furthermore, interactive lecture requires more time and coordination such that effectiveness of classes with fewer students is suggested to be evaluated. Moreover, appropriate performance along with paper-pencil tests is suggested to evaluate the learning outcomes of the interactive teaching method.

 This study has several limitations. First, the number of samples is low; therefore, generalization of the results is difficult. Second, some of the results are based on the participants' own statements that they may not have high credibility. Although the issue of whether students are or not legitimate referees to evaluate teaching methods was considered by the experts, there is a general sense that students are reliable evidence for research in education.(30) They are able to report the learning experience that can be invaluable, satisfactory and useful, and to express the effectiveness of the teaching method and the quality of the teacher’s interaction with them.(31) Third, the increase in knowledge in this study is related to short-term results, and knowledge retention in the long term has not been investigated. Therefore, it is suggested that further studies be conducted with more samples. Additionally, in future studies, the impact of other interactive methods can be compared. It is suggested that the consequences of the Kirkpatrick’s higher levels be investigated after applying new methods. In the interactive lecture method, owing to using discussion in small groups, it is anticipated that critical thinking and problem-solving skills will be improved. Accordingly, future studies can examine the impact of such methods on changing these skills.

## Conclusion

Outcomes of the interactive lecture have been more desirable than traditional lecture to teach the credit of intensive nursing care. However, it needs more time compared to the traditional lecture. Moreover, interactive lectures are likely to increase students' satisfaction and stimulate their learning. Applying active teaching methods can help to achieve educational objectives. More evidence is needed to draw a more solid conclusion on these issues.
